# Sexual Dimorphism in Tuberculosis Incidence: Children Cases Compared to Adult Cases in Tuscany from 1997 to 2011

**DOI:** 10.1371/journal.pone.0105277

**Published:** 2014-09-25

**Authors:** Alessia Stival, Elena Chiappini, Carlotta Montagnani, Elisa Orlandini, Carlotta Buzzoni, Luisa Galli, Maurizio de Martino

**Affiliations:** 1 Department of Health Sciences, University of Florence, Anna Meyer Children’s University Hospital, Florence, Italy; 2 Information Technology Section, Tuscany Regional Government Department of Right to Health and Solidarity Policies, Florence, Italy; 3 Msc. Clinical and Descriptive Epidemiology Unit, Institute for cancer study and prevention ISPO, Florence, Italy; Institut de Pharmacologie et de Biologie Structurale, France

## Abstract

**Background:**

In most countries, men seem to be more susceptible to tuberculosis (TB) than women, but only few studies have investigated the reasons of this gender incidence difference. The effect of sexual hormones on immunity is possible.

**Methods:**

Data from children and adults, living in Tuscany, hospitalized for TB in all the thirty-one regional hospitals from January 1^st^ 1997 to December 31^st^ 2011, were analyzed using the International Classification of Disease, 9^th^ Revision, Clinical Modification.

**Results:**

During the study period, 10,744 patients were hospitalized with TB diagnosis, precisely 279 (2.6%) children [0–14 years], 205 (1.9%) adolescents [15–18 years] and 10,260 (95.5%) adults [≥18 years]. The male population ranged from 249 patients (51.4%) in children and adolescents, to 6,253 (60.9%) in adults. Pulmonary TB was the most common form both in children and adults. Men were more likely than women to have pulmonary TB after puberty, while no significant differences were found between males and females in the hospitalized children. The male gender also resulted the most affected for the extra-pulmonary disease sites, excluding the lymphatic system, during the reproductive age.

**Conclusions:**

Our findings suggest a possible role of sexual hormones in the development of TB. No significant male-female difference was found in TB incidence among children, while a sex ratio significantly different from 1∶1 emerged among reproductive age classes. An increased incidence difference also persisted in older men, suggesting that male-biased risk factors could influence TB progression. Some limitations of the study are the sample size, the method of discharge diagnosis which could be deficient in accuracy in some cases, the increasing number of immigrants and the lack of possible individual risk factors (smoke and alcohol). Further studies are needed to investigate the possible hormone-driven immune mechanisms determining the sexual dimorphism in TB.

## Introduction

Susceptibility to some infectious diseases is affected by gender [1], [2]. Particularly, in most countries, men seem to be more prone to tuberculosis (TB) than women, although no underlying factors have been identified yet [3]. Gender-specific susceptibility is influenced by multiple factors, such as socioeconomic and cultural (for example women are more inclined to have a help-seeking behaviour in Western countries, while they meet barriers in accessing health care services in developing countries), but also on immunological and endocrine factors [3], [4], [5]. Despite the evidence of gender specific differences in infections caused by all kinds of pathogens (bacteria, fungi, protozoan parasites and viruses), only few studies have investigated the sexual dimorphism in TB [1], [2], [6].

A 1998 review analysed sex differences in the epidemiology of TB in industrialized countries from the middle of the last century to the 1990 s, leading to the important observation that TB rates in males were higher than those in females after the age of 15, even if there was evidence that progression from infection to disease in young to early-middle-aged women was more frequent than in older ones [7]. In a multicentre case-control study conducted in three West African countries from 1999 to 2001, the male gender resulted an independent risk factor for TB [8]. A recent analysis of sex bias in infectious disease epidemiology in the Brazilian population found that male-female incidence rate ratios were significantly different after puberty for pulmonary TB (PTB) [9]. According to these studies, the male-female ratio of TB in countries belonging to the World Health Organization (WHO) was 1.7 globally, ranging from 1.1 to 2.2, in 2011 [10].

Few recent epidemiological studies on paediatric TB in Europe reported TB incidence by gender. Authors did not find any differences among male and female incidence and this finding has not been discussed extensively [11], [12], [13]. To our knowledge, only one recent study analysing 1,370 cases of paediatric TB in London, between 1999 and 2006, described a higher TB incidence in girls [14], but the authors did not report if this difference was statistically significant.

Aiming to investigate a role for sexual hormones in TB susceptibility and disease outcome between men and women, we analysed data from children and adults hospitalized for TB in Tuscany over a period of 15 years, focusing our attention on gender differences in all age classes. In order to avoid differences in care-seeking behaviour, at first the study population was considered in its totality and then differentiated in natives and immigrants. To discuss possible confounding factors, such as smoking and alcohol, the main sites of disease were studied separately.

## Methods

### Data sources

We created a database with the total number of hospitalizations for TB in all the thirty-one Tuscan hospitals from January 1st, 1997 to December 31st, 2011. We identified all patients encoded with International Classification of Disease, 9th Revision, Clinical Modification (ICD-9-CM) codes 010–018, who were discharged from a Tuscan hospital with TB diagnosis. Patients living outside Tuscany were excluded. We also performed a double-check, using personal data of the patients, to avoid a possible duplication in reporting cases if someone was transferred from a hospital to another one. We obtained the number of children and adults living in Tuscany during the study period, referred to each year and allocated by gender and age class, by the Italian National Statistical Institute database. The Tuscan population was classified in five age classes, according with the main periods of human life (childhood and adolescence – post-pubertal and reproductive years – advanced adulthood and old age).

TB cases were classified as either PTB or extra-pulmonary tuberculosis (EPTB), according with the site of disease. We considered as EPTB all the follow TB forms: intestinal, lymphatic, miliary, bone, kidney TB, TB of the central nervous system and of other sites (including spleen, ear, eye, pericardium and adrenal glands). Not specified cases were reported but excluded from our analysis.

### Statistical analysis

We estimated the female-to-male incidence rate ratios (IRRs) and their 95% CI for the five age classes in the whole study population. TB incidence rates were calculated as cases per 100,000 person/years. A Poisson regression model was used to evaluate changes in incidence rate ratios for age class, calendar period at diagnosis, gender and nationality in the study population from 2003 to 2011. Our dependent variables were age (0-14/15-24/25-44/45-64 and 65+ years), gender (male/female), period (2003–2006/2007+) and nationality (Italian/immigrant). We selected as additive terms age, gender, period and nationality and interaction between age and sex. We elaborated our data with STATA software version 12.

### Ethics statement

The study received approval from the Ethics Committee of Anna Meyer Children’s University Hospital. Data were obtained from an electronic database using ICD-9-CM without written informed consent, being a descriptive epidemiological study with no human experimentation. This does not constitute a privacy violation because when someone is hospitalized gives consent for being included in the national database. Furthermore, patient information was anonymized and de-identified prior to analysis.

## Results

During the study period, overall 10,744 patients were hospitalized with TB diagnosis in the thirty-one Tuscan hospitals, specifically 279 (2.6%) children [0–14 years], 205 (1.9%) adolescents [15–18 years] and 10,260 (95.5%) adults [≥18 years]. Paediatric patients were admitted to the Anna Meyer Children’s University Hospital in Florence for the most part (exactly 233 patients, that corresponded to 48.1%). Among children and adolescents, 249 (51.4%) were males. In the group of adults, 6,253 (60.9%) were males. The majority of adults (n = 4,307; 42.0%) were admitted to three main Tuscan hospitals (Florence, Massa-Carrara and Arezzo). Globally, 3,202 (29.8%) patients were immigrants.

**Figure 1:**
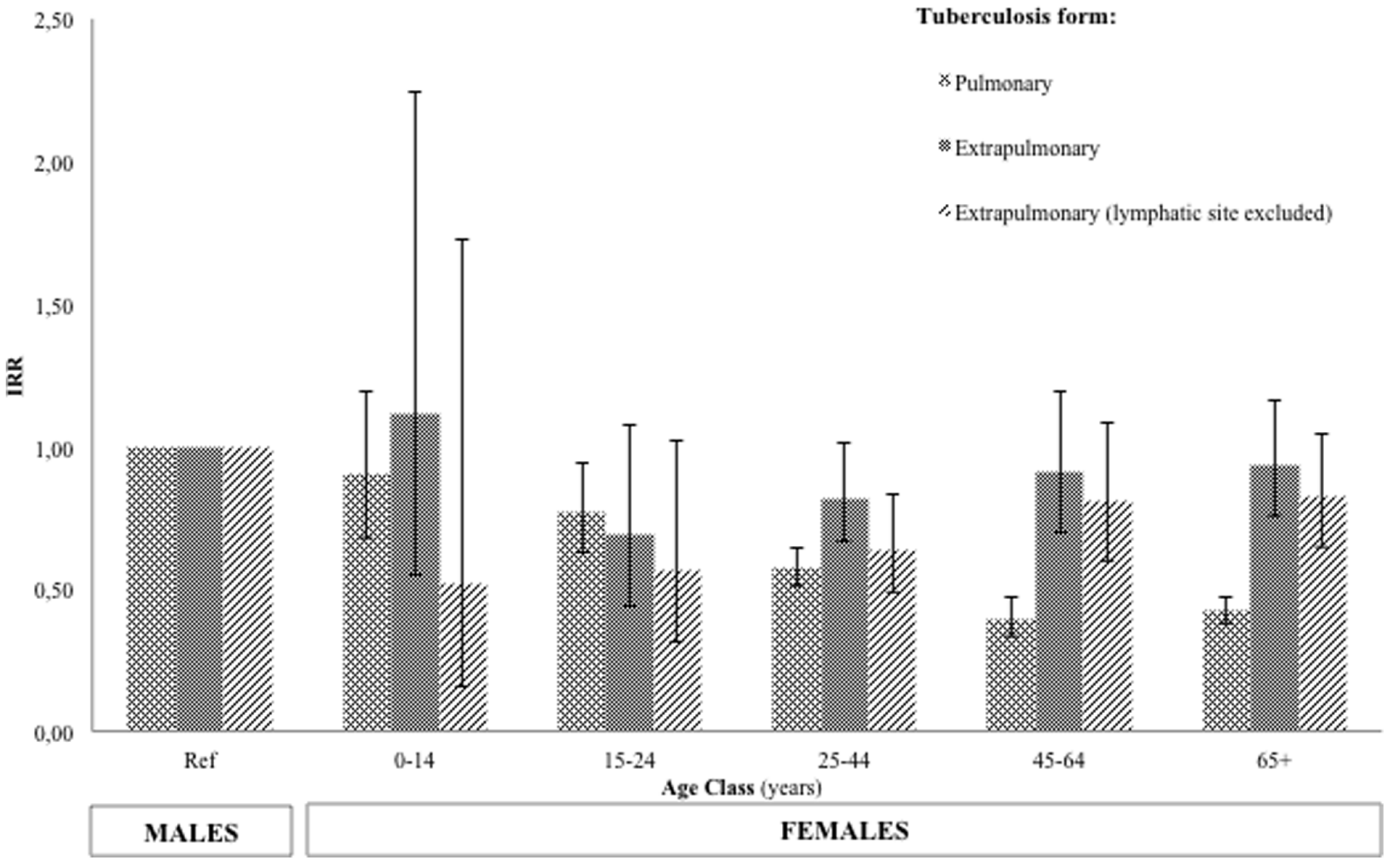
Female incidence rate ratios for each age group (using male gender as a reference) in all the observed population (2003–2011).

**Figure 2:**
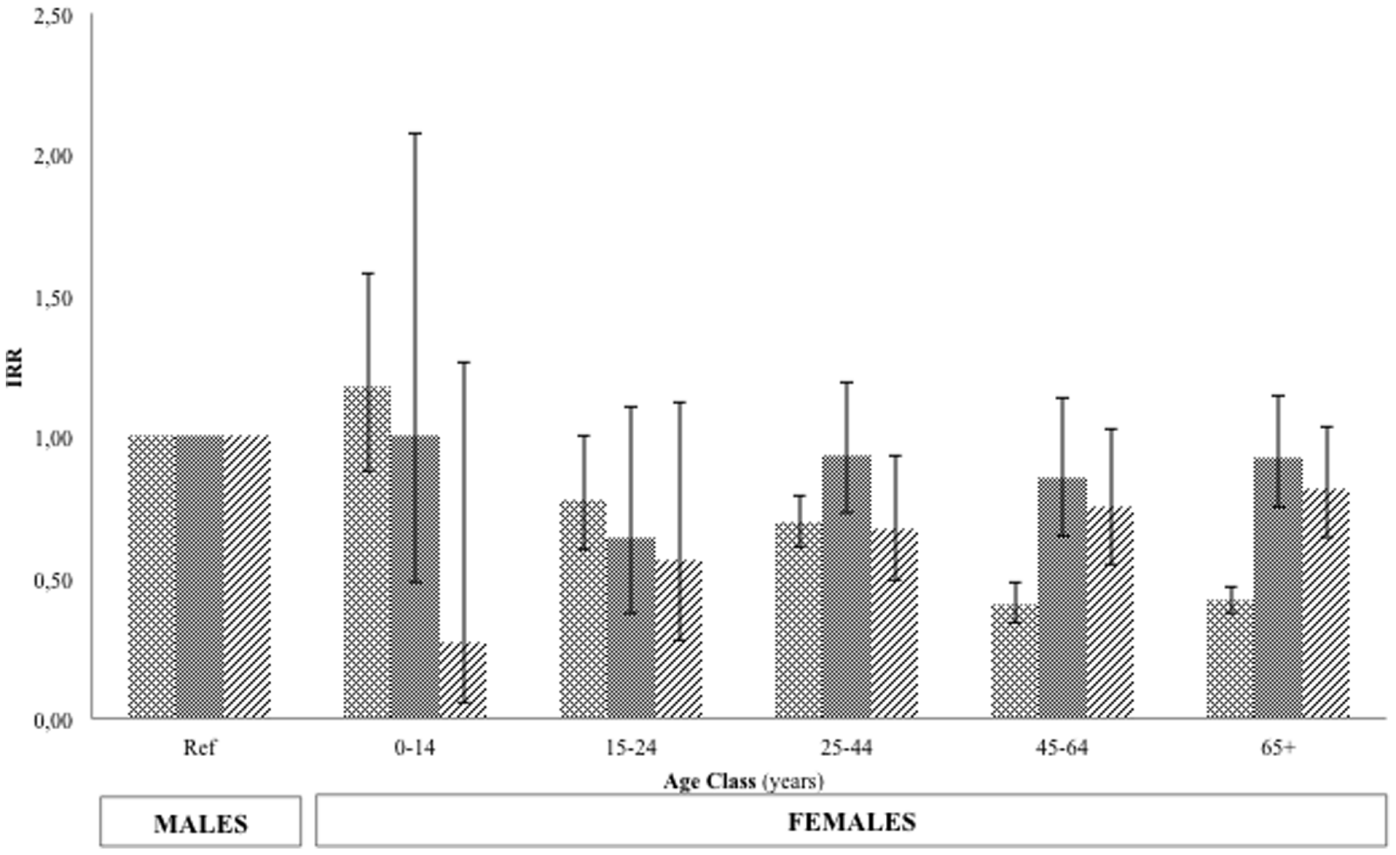
Female incidence rate ratios for each age group (using male gender as a reference) in the Italian observed population (2003–2011).

No significant differences in TB incidence were found between males and females in the hospitalized children (Table 1 and 2). The observed TB incidence in males and females started to differ significantly from the age of 25 years (IRR 1.633, 95% CI 1.513–1.763). From this age onwards, men were more likely than women to develop the disease. Using Poisson regression model, a significant difference in PTB incidence between males and females emerged after puberty [female-to-male IRR 0.77; 95% CI: 0.63–0.94] (Table 3). We also compared disease IRRs in the whole study population (Figure 1) to those of the native Tuscan patients, excluding the immigrants (Figure 2). The conclusion was that the trend was similar.

**Table 1:** Cases of tuberculosis in Tuscan population, 1997–2011: age-stratified female:male annual incidence rate ratios and 95% confidence intervals.

Age class (years)	Population (N)*		TB cases (N)		Annual incidence rate**		Female to male incidence rate (IRR)	IRR 95% confidence interval
	Males	Females	Males	Females	Males	Females		
0–14	221,748	209,613	195	183	5.86	5.82	0.993	0.812–1.750
15–24	171,756	163,204	402	351	15.60	14.34	0.919	0.796–1.527
25–44	531,909	522,027	1,752	1,053	21.96	13.45	0.612	0.567–0.661
45–64	469,637	494,350	1,402	728	19.90	9.82	0.493	0.451–0.539
65+	338,496	474,937	2,751	1,927	54.16	27.05	0.499	0.471–0.529

* Average annual population during the analysed period.

** TB incidence rates were calculated as cases per 100,000 population.

**Table 2:** Tuberculosis cases in the Tuscan population differentiated by disease site and gender (1997–2011).

TUBERCULOSIS SITES - MALES										
Age class	Pulmonary	Extra-pulmonary							Not Specified	Total cases
years	n	Intestinal n	Lymphatic n	Miliary n	Bone n	Kidney n	CNS n	Others n	n	n
**0–14**	141		20	3	2		5		24	195
**15–24**	304	8	29	5	15		6		35	402
**25–44**	1278	23	96	40	64	15	48	5	183	1752
**45–64**	1054	10	25	26	45	42	31	5	164	1402
**65+**	2186	21	45	23	54	66	29	13	314	2751
	**4963**	**62**	**215**	**97**	**180**	**123**	**119**	**23**	**720**	**6502**
**TUBERCULOSIS SITES - FEMALES**										
**Age class**	**Pulmonary**	**Extra-pulmonary**							**Not Specified**	**Total cases**
**years**	**n**	**Intestinal n**	**Lymphatic n**	**Miliary n**	**Bone n**	**Kidney n**	**CNS n**	**Others n**	**n**	**n**
**0–14**	138	1	23		2		1		18	183
**15–24**	253	5	35	6		1	9	1	41	351
**25–44**	699	20	103	27	25	19	24	8	128	1053
**45–64**	423	5	44	8	28	56	54	3	107	728
**65+**	1318	13	147	35	69	51	39	10	245	1927
	**2831**	**44**	**352**	**76**	**124**	**127**	**127**	**22**	**539**	**4242**

**Table 3:** Poisson regression model to evalute incidence rate ratio for age class, gender, period of diagnosis and nationality (2003–2011).

		Pulmonary TB					Extra-pulmonary TB					EPTB excluded lymphatic TB				
Variable		n	IRR	95% CI		p-value	n	IRR	95% CI		p-value	n	IRR	95% CI		p-value
**Age (years)**	**0–14 (ref.)**	195	1.00				31	1.00				12	1.00			
	**15–24**	381	1.93	1.55	2.39		80	5.44	3.22	9.19		48	7.35	3.61	14.99	
	**25–44**	1,332	0.93	0.73	1.19		355	3.79	2.21	6.50		232	6.34	3.08	13.05	
	**45–64**	661	2.56	2.07	3.18		213	7.41	4.35	12.61		177	12.04	5.88	24.64	
	**65+**	1,298	0.90	0.68	1.19	<0.001	342	1.11	0.55	2.25	<0.001	269	0.52	0.16	1.72	<0.001
**Gender**	**Male (ref.)**	94	1.00				15	1.00				8	1.00			
**0–14 years**	**Female**	101	0.90	0.68	1.19	0.4540	16	1.11	0.55	2.25	0.7710	4	0.52	0.16	1.72	0.2840
	**Male (ref.)**	218	100				48	1.00				31	1.00			
**15–24 years**	**Female**	163	0.77	0.63	0.94	0.0110	32	0.69	0.44	1.08	0.1020	17	0.56	0.31	1.02	0.0580
	**Male (ref.)**	854	1.00				197	1.00				143	1.00			
**25–44 years**	**Female**	478	0.57	0.51	0.64	0.0000	158	0.82	0.66	1.01	0.0630	89	0.64	0.49	0.83	0.0010
	**Male (ref.)**	471	1.00				110	1.00				97	1.00			
**45–64 years**	**Female**	190	0.39	0.33	0.47	0.0000	103	0.91	0.70	1.19	0.4910	80	0.80	0.60	1.08	0.1490
	**Male (ref.)**	819	1.00				149	1.00				126	1.00			
**65+ years**	**Female**	479	0.42	0.38	0.47	0.0000	193	0.94	0.76	1.16	0.5460	143	0.82	0.65	1.04	0.1070
**Period**	**2003–2006**	1,851	1.00				527	1.00				392	1.00			
	**2007+**	2,016	0.79	0.75	0.85	0.0000	494	0.69	0.61	0.78	0.0000	346	0.65	0.56	0.75	0.0000
**Nationality**	**Italian**	3,195	1.00				873	1.00				629	1.00			
	**Immigrant**	672	3.34	3.06	3.65	0.0000	148	2.85	2.37	3.42	0.0000	109	3.25	2.62	4.03	0.0000

We did not find any statistically significant differences in the incidence rates of both PTB and EPTB between male and female children [female-to-male IRR for PTB: 1.035; 95% CI: 0.819–1.309; female-to-male IRR for EPTB: 0.952; 95% CI: 0.556–1.601]. Considering all the age classes, no characteristic gender bias was highlighted for EPTB (Table 3).

Among extra-pulmonary disease sites, the lymphatic system was the most represented (Table 2). If considered alone, a significant difference was found in the incidence rates between male and female adults, with a predominance of cases in women. The multivariate analysis, once the patients with lymphatic TB were excluded from the extra-pulmonary cases, showed a statistically significant male-female difference in the reproductive age and the male gender was the most affected (Table 3).

## Discussion

In the last two decades, very few studies analysed trends in childhood TB considering the gender of patients [11], [12], [13], while more authors studied sex bias in TB epidemiology among adults, underlining gender differences in TB incidence [3], [9], [15]. We did not observe any significant male-female difference in TB incidence among children. After puberty and overall after the age of 25 years, we found a consistent discrepancy, in line with a study reporting sex-specific TB incidence rate in San Francisco population, from 1991 to 1996 [16].

Our findings may suggest a role of sex hormones in TB outcome. We observed an initial difference in male and female TB incidence after puberty, but difference was more consistent after 25 years of age. Length of exposure period to sex hormones could influence the immune response against TB, as suggested by other models including pregnancy puberty or ear and nose hair [17], [18], [19], [20].

There are several evidences that androgens and estrogens play an important role in gender immunological dimorphism: they modulate the immune system controlling synthesis of pro-inflammatory and immunosuppressive cytokines, Toll-like receptor (TLR) expression on cell surface, skewing of the inflammatory response (T_H_1 vs. T_H_2) and antibody production [1], [2], [21], [22], [23], [24], [25].

The immune-modulating role of estrogens remains an unresolved paradox because their effects on cytokine secretion depend on cell types, host conditions and estrogen concentrations [26], [27], [28], [29]. However, estrogens have overall an activator effect on the immune system, since they promote T_H_2-response development, which induces the production of IL-4, IL-5 and IL-10 [4].

Some authors affirm that sex hormones do not explain completely sexual dimorphism and that innate differences exist between men and women in their innate and adaptive immune responses [2], [6], [28], [30], [31], [32], [33] and, consequently, a strong relationship between gender immune differences and the specific immune response to different pathogens [2].

Unfortunately, the absence of measurable differences between child boys and girls does not provide any critical evidence for or against a role for behavioural versus hormone-related risk factors because these variables start to differ roughly after childhood.

In our study, an increased incidence difference between men and women also persisted after menopause. It is possible that male-biased risk factors, such as smoking and alcohol intake, could influence TB progression in older men [7], [9], [34]. It is known that the percentage of smokers is higher among men in Tuscany, even if it is not as high as the national median; furthermore, incidence of smokers among Tuscan women is greater than the national median [35]. A consistent number of studies have analysed the association between smoking and TB, demonstrating a causal relationship between the exposure to tobacco smoke, both passive and active, and the disease [34], [36], [37].

Regarding alcohol intake, a systematic review has investigated the strength of its association with TB, finding that the risk of active TB was substantially elevated in people who drink than 40 g alcohol per day, and/or have an alcohol use disorder [38]. In the case-control study these addictions were associated with acquired multi-drug resistance TB cases in multivariate analysis [37]. Supposing a higher abuse of alcohol among the male sex, we consider it as a possible immunosuppressive factor in older male patients, together with the habit of smoking.

To find, using Poisson regression model, that male adults were also more affected by EPTB (excluded lymphatic TB) than women during the reproductive age reinforced the hypothesis that gender-related behaviour was not sufficient to explain such a difference. By our analyses only the lymphatic TB among the EPTB forms was more common in women, in partial agreement with findings previously reported in literature [39], [40], [41]. However, the number of lymphatic TB cases was too small to perform any powerful test and limited us in further discussions.

Our study has some limitations. First of all, TB cases were identified on the base of discharge diagnosis code ICD-9, a simple but useful method to obtain epidemiological information, but the accuracy of some TB diagnosis might be questionable. However, recently, other Italian studies used this means to elucidate TB epidemiology in children and adults [42], [43], [44], [45], [46]. Moreover, our results might be influenced by increasing number of immigrants who come in our country and may contribute to widen just those age classes in which we noted an increased TB incidence and a major difference between the sexes. We could not estimate TB incidence in the immigrant population in Tuscany from 1997 to 2003, since no official numbers of regularly registered immigrants are available for the study period. Reducing the study period in the Poisson regression model, we had the possibility to perform a more accurate evaluation of TB incidence distinguishing the Italian cases from the immigrant ones. However, immigrants without a legal residence permit were excluded. It is also possible that some Italian patients, resident in Tuscany, may have been admitted to extra-regional hospitals. The influence of some risk factors, like alcohol and tobacco smoke, can be only supposed because these data were not available in our database.

In conclusion, our findings suggest (but not confirm) a possible role of sexual hormones in the development of TB disease. Gender should be considered not only from an epidemiological point of view but also from a clinical one, being an important factor in the pathogenesis, management and prognosis of this infection. Further studies on gender dimorphism in TB disease are needed in order to develop therapeutic strategies against this disease, which continues to claim millions of people in the world.
